# Decreased RIPK1 expression in chondrocytes alleviates osteoarthritis via the TRIF/MyD88-RIPK1-TRAF2 negative feedback loop

**DOI:** 10.18632/aging.102354

**Published:** 2019-10-11

**Authors:** Shuang Liang, Zheng-Gang Wang, Zhen-Zhen Zhang, Kun Chen, Zheng-Tao Lv, Yu-Ting Wang, Peng Cheng, Kai Sun, Qing Yang, An-Min Chen

**Affiliations:** 1 Department of Orthopedics, Tongji Hospital, Tongji Medical College, Huazhong University of Science and Technology, Wuhan 430030, China; 2 Department of Rehabilitation Medicine, The Third Affiliated Hospital of Southern Medical University, Guangzhou 510000, China; 3 Department of Orthopedics, The First Affiliated Hospital of USTC, Division of Life Sciences and Medicine, University of Science and Technology of China, Hefei 230001, Anhui, P.R. China; 4 Department of Oral Medicine, Infection and Immunity, Harvard School of Dental Medicine, Boston, MA 02115, USA

**Keywords:** RIPK1, TRIF, MYD88, TRAF2, osteoarthritis

## Abstract

Osteoarthritis (OA) is the most common degenerative joint disease and involves the loss of articular cartilage integrity, formation of articular osteophytes, remodeling of subchondral bone, and synovitis. Knockdown of receptor interacting serine/threonine kinase (RIPK) 1 leads to anti-inflammatory and anti-apoptotic effects. However, the involvement of RIPK1 in the pathogenesis of OA is unclear. Here, we evaluated the effect of RIPK1 on chondrocytes and elaborated the underlying molecular mechanism. Knockdown of RIPK1 protected chondrocytes against inflammation and apoptosis induced by interleukin (IL)-1β *in vitro* and *in vivo*. RIPK1 was required for myeloid differentiation primary response 88 (MyD88)- and TIR-domain-containing adapter-inducing interferon b (TRIF)-mediated production of matrix metalloproteinases (MMPs) in OA. Moreover, overexpression of RIPK1 promoted the expression of tumor necrosis factor receptor-associated factor 2 (TRAF2), which blocked the expression and phosphorylation of RIPK1. Upregulation of TRAF2 decreased the expression of TRIF, MyD88, and MMPs in chondrocytes. Furthermore, knockdown of RIPK1 blocked activation of the nuclear factor-κB (NF-κB) and c-Jun N-terminal kinase (JNK) signaling pathways. In summary, knockdown of RIPK1 alleviated OA in a manner mediated by the TRIF/MyD88-RIPK1-TRAF2 negative feedback loop and activation of the NF-κB and JNK signaling pathways.

## INTRODUCTION

Osteoarthritis (OA) is the most prevalent progressive and degenerative joint disease, and is characterized by alternations in the morphology, composition, and integrity of the articular cartilage, in addition to synovial inflammation [1, 2]. OA causes pain in the hands, knees, hips, and spine, reducing quality of life and imposing a considerable socioeconomic burden [3]. A variety of factors are related to the progression of OA, including age, gender, heredity, joint injuries, diet, and obesity [4]. Also, the increased production of proinflammatory factors, such as interleukin (IL)-1β, tumor necrosis factor (TNF)-α, IL-6, IL-15, IL-17, and IL-18, in cartilage, bone, and synovium upregulates the expression of matrix metalloproteinases (MMPs) [5–7]. The molecular mechanism of OA, however, is poorly understood.

Previous studies showed that blocking of TLR signaling down-regulates cartilage catabolism in vitro, and could protect animals from experimental OA [8, 9]. TLRs are pattern-recognition receptors that recognize pathogen-associated microbial patterns, leading to activation of nuclear factor-κB (NF-κB) and interleukin regulatory factor 3 [10]. Upon ligand binding, receptor-interacting serine 1 (RIPK1) is recruited to IL-1R/TLR superfamily complexes via the death domain (DD), which influences cell survival and proinflammatory signaling [11]. Moreover, RIPK1 interacts with TNF receptor 1 (TNFR1) through its DD and DD-containing adaptor TNFR type 1-associated death domain protein (TRADD), forming complex I [12]. Complex I, composed of RIPK1, TRADD, the adaptor protein TNF receptor-associated factor 2 (TRAF2), and the ubiquitin ligases cellular inhibitor of apoptosis protein 1 (cIAP1) and cIAP2, activates the mitogen-activated protein kinase (MAPK) and NF-κB signaling pathways via a complex series of ubiquitination events [13]. After de-ubiquitination of RIPK1, RIPK1, the DD-containing adaptor Fas-associated protein with death domain (FADD), TRADD, and caspase-8 translocate to the cytosol as complex II and trigger cell-death signaling [14, 15]. Additionally, in response to severe DNA damage, complex II can form complex IIb, in which FADD is directly oligomerized by RIPK1 to recruit caspase-8 and FLIP [16]. The recruitment of caspase-8 to complex IIb initiates apoptosis [17]. These findings suggest a potential mechanism for RIPK1 in regulating OA. However, further studies are needed to clarify the exact effect of RIPK1 on the pathogenesis of OA. Moreover, TRAF2 interacts with RIPK1 directly via its N-terminus [18, 19]. TRAF2 plays an important role in alleviating apoptosis and inflammation [20], and knockout of TRAF2 triggers apoptosis and inflammation by an NF-κB–independent mechanism [21]. Furthermore, exposure of primary cells to IL-1β or TNF-α causes overexpression of TRAF2, suggesting that TRAF2 regulates IL-1R/TLR signaling [22, 23]. Although TRAF2 regulates TNFR signaling, it is also involved in other signaling pathways, such as the TLR/IL-1R signaling pathway [24]. The mitochondrial adaptor protein mitochondrial antiviral signalling protein (MAVS) and the mitochondrial E3 ligase mitochondrial ubiquitin ligase activator of NF-κB (MULAN)] recruit TRAF2 to mitochondria, which promotes an inflammatory response [25].

To our knowledge, the role of RIPK1 in OA has not been reported to date. We hypothesized that RIPK1 knockdown would exert an anti-inflammatory effect on IL-1β–treated chondrocytes and alleviate OA in a mouse model. In this study, we used mice with RIPK1 knockdown in the articular cartilage to generate OA model. We found that the mice with RIPK1 knockdown alleviated cartilage degeneration and synovial inflammation. Conversely, overexpression of RIPK1 in the chondrocytes enhanced catabolism. We also investigated the role of TRAF2 in OA. Our findings suggest that RIPK1 is a potential target for the treatment of OA.

## RESULTS

### The phosphorylation level of RIPK1 is increased in mouse knee articular cartilage

**Figure 1:**
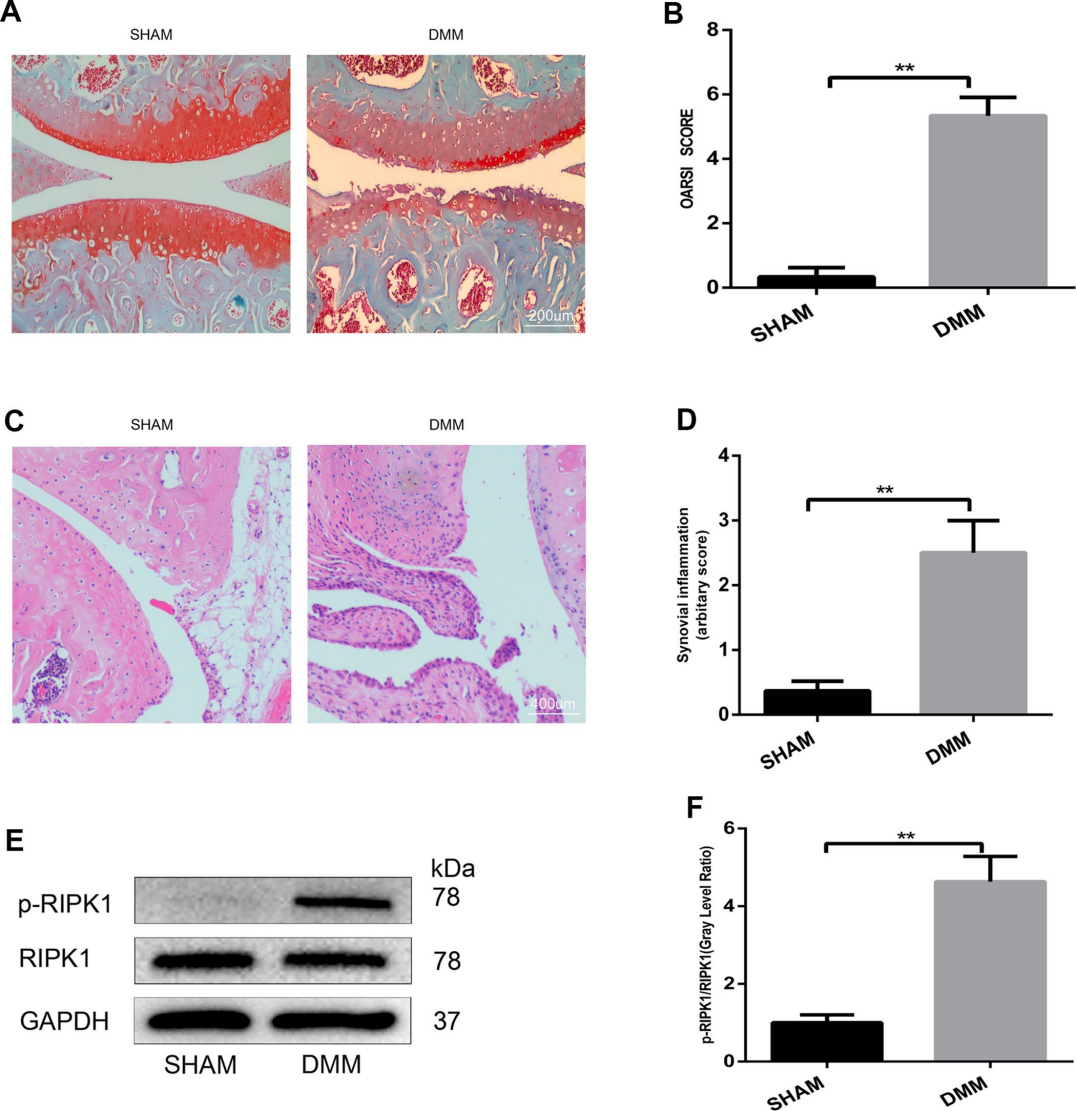
**The phosphorylation level of RIPK1 is increased in mouse knee articular cartilage.** (**A**, **B**) Safranin O/Fast Green-stained sagittal-plane images of tibial and femoral cartilage from the sham and destabilized medial meniscus (DMM) groups; the Osteoarthritis Research Society International (OARSI) score was significantly increased in the DMM group (n = 10); scale bar = 200 μm. (**C**, **D**) Representative hematoxylin and eosin (HE)-stained images and synovial inflammation scores in the sham and DMM groups (n = 10); scale bar = 400 μm. (**E**, **F**) Western blots and quantitative data of p-RIPK1 in the sham and DMM groups. The experiments were repeated three times independently. Columns represent means ± SD. **p < 0.01.

To our knowledge, no study has assessed the role of RIPK1 in articular cartilage destruction. To explore whether RIPK1 is involved in the pathogenesis of OA, we evaluated RIPK1 phosphorylation in the knee articular cartilage of mice with OA. Safranin O/Fast Green staining (Figure 1A) was performed to assess structural changes, fibrillations, and vertical clefts of cartilage in the DMM and sham groups. The Osteoarthritis Research Society International (OARSI) score (Figure 1B) was significantly higher (5 points; p < 0.01) in the DMM group than the sham group (0.5 points). Also, synovial inflammation was more severe (arbitrary score, 2.5 points) in the DMM group than the sham group (0.3 points) (p < 0.01; Figure 1C, 1D). The OARSI and arbitrary scores were determined by three blinded observers to improve reliability. Next, we examined RIPK1 phosphorylation in articular cartilage of the DMM and sham groups by Western blotting. The phosphorylation of RIPK1 in the knee articular cartilage of DMM mice was significantly upregulated (Figure 1E, 1F). *In vitro*, the phosphorylation of RIPK1 was significantly increased in chondrocytes treated with IL-β for 10 min (Figure 3H and 3I). Taken together, these results suggest a role for RIPK1 in OA.

### Knockdown of RIPK1 attenuates cartilage destruction *in vivo*

**Figure 2:**
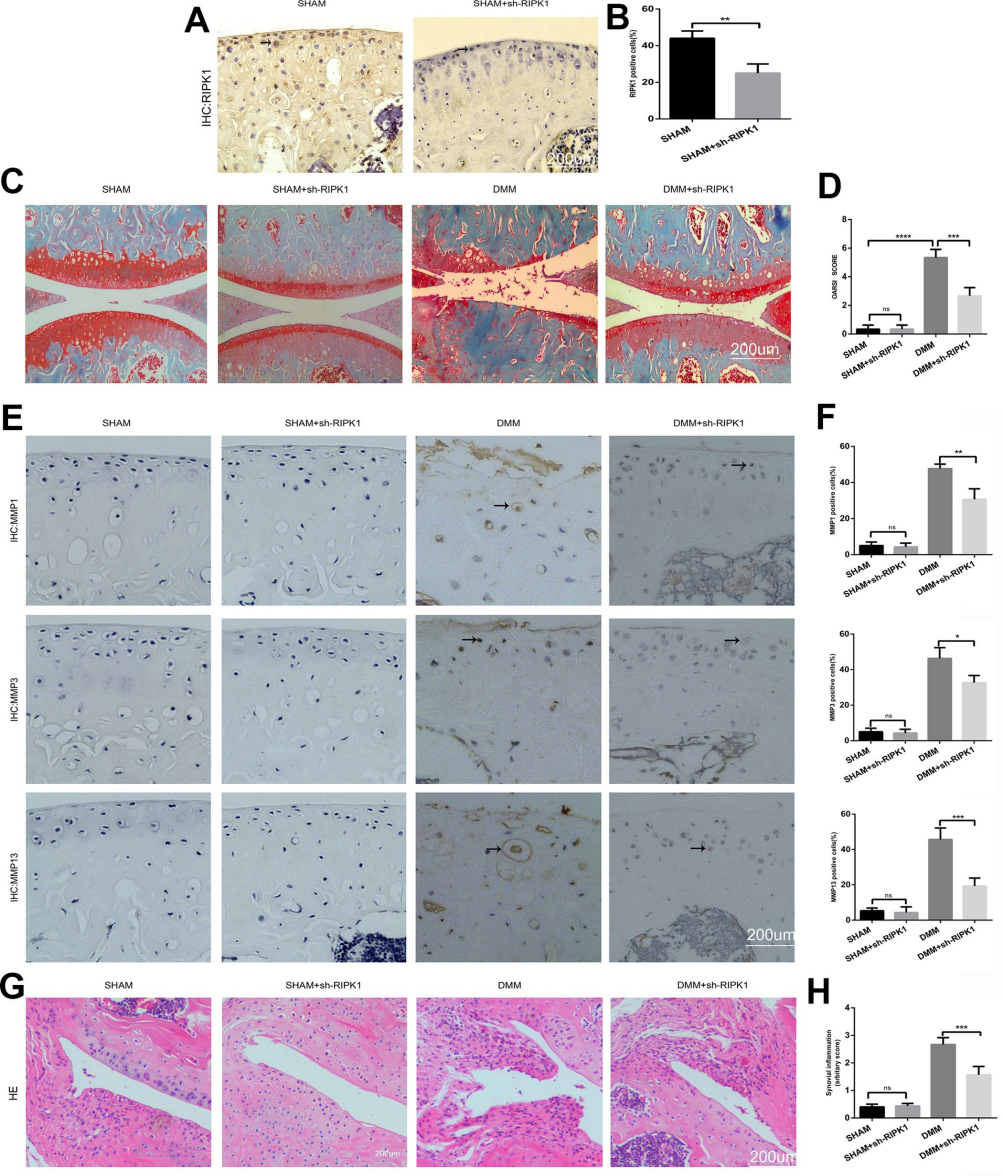
**Knockdown of receptor interacting serine/threonine kinase (RIPK) 1 attenuates cartilage degeneration *in vivo*.** (**A**, **B**) Immunohistochemical staining of RIPK1 in mice transfected with Ad-NC and Ad-shRIPK1 (n = 10); scale bar = 200 μm. (**C**, **D**) Safranin O/Fast Green-stained articular cartilage; the degree of cartilage degeneration was evaluated by calculating the OARSI score (n = 10); scale bar = 200 μm. (**E**, **F**) Immunohistochemical staining of matrix metalloproteinases (MMPs; n = 10); scale bar = 200 μm. (**G**, **H**) Representative HE-stained images and synovial inflammation scores (n = 10); scale bar = 200 μm. Columns represent means ± SD. *p < 0.05, **p < 0.01, ***p < 0.001, ****p < 0.0001.

To confirm the role of RIPK1 in OA, mice underwent intra-articular injection of Ad-shRIPK1 1 week after DMM or sham surgery. Eight weeks later, immunohistochemical, hematoxylin and eosin (HE), and Safranin O/Fast Green staining was performed to assess the severity of OA. Immunohistochemical staining showed that RIPK1 expression was decreased in the mice administered Ad-shRIPK1 (Figure 2A, 2B; p < 0.01). Compared with the sham group, in the DMM group the tibial and femoral articular cartilage showed obvious destruction, including erosion, fibrillations, and loss of cellularity. In contrast, Ad-shRIPK1 significantly ameliorated cartilage destruction, based on the OARSI scores (Figure 2C, 2D). The severity of synovitis showed the same trend as the OARSI score (Figure 2G, 2H). The protein levels of MMP1, MMP3, and MMP13 in articular cartilage were markedly decreased in the DMM+sh-RIPK1 group compared with the DMM group (Figure 2E and 2F). Therefore, knockdown of RIPK1 exerts a significant protective effect against OA *in vivo*.

### Knockdown of RIPK1 inhibits IL-1β–induced catabolism and production of proinflammatory cytokines *in vitro*

**Figure 3:**
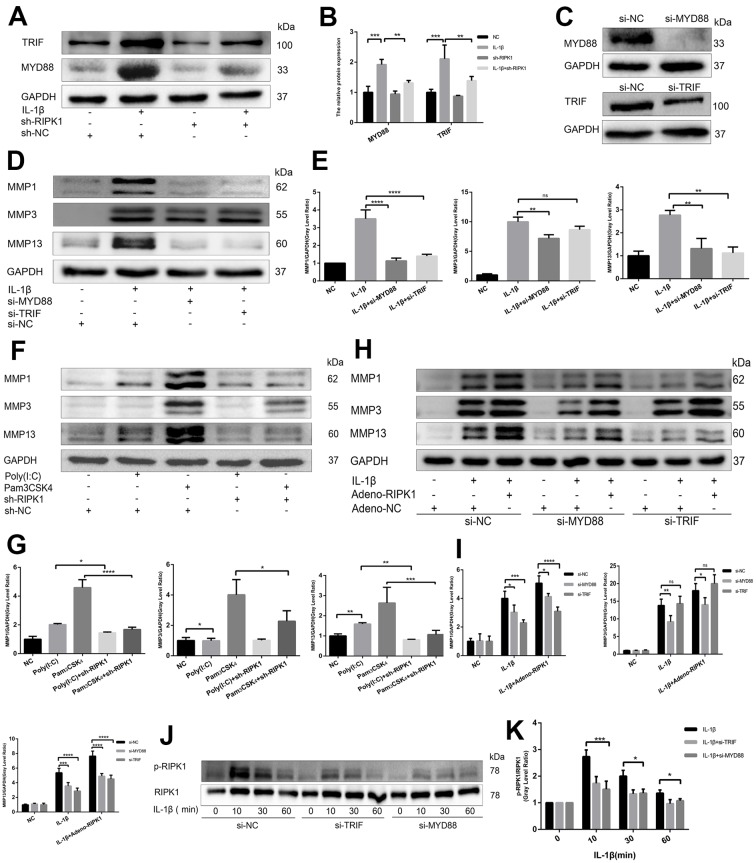
**Knockdown of RIPK1 inhibits interleukin (IL)-1β–induced production of catabolic enzymes and proinflammatory cytokines *in vitro*.** (**A**, **B**) Representative western blots and quantitative data of RIPK1 and p-RIPK1 in chondrocytes transfected with Ad-shRIPK1 and Ad-RIPK1. (**C**, **D**) Knockdown of RIPK1 reduced the IL-β–induced expression of MMPs in chondrocytes. (**E**, **F**) Overexpression of RIPK1 promoted the IL-β–induced expression of MMPs in chondrocytes. (**G**) Enzyme-linked immunosorbent assay (ELISA) of the tumor necrosis factor (TNF)-α level in culture supernatants. (**H**, **I**) Western blots and quantitative data of p-RIPK1 in chondrocytes treated with IL-1β. The experiments were repeated three times independently. Columns represent means ± SD. *p < 0.05, **p < 0.01, ***p < 0.001, ****p < 0.0001.

To evaluate the role of RIPK1 in the production of catabolic enzymes and proinflammatory cytokines, RIPK1 was knocked down using Ad-shRIPK1 or increased using an adenovirus overexpressing RIPK1. Western blotting confirmed the expression of RIPK1 and p-RIPK1 (Figure 3A, 3B). MMPs are key regulators of the destruction of articular cartilage [26]. Chondrocytes were infected with the adenovirus for 2 days and treated with IL-β for 2 days. Western blotting indicated that IL-β increased, but RIPK1 knockdown decreased, the production of MMP1, MMP3, and MMP13 (Figure 3C, 3D). In contrast, the overexpression of RIPK1 increased the IL-β–induced expression of MMPs in chondrocytes (Figure 3E, 3F). TNF-α, a proinflammatory cytokine, plays an essential role in the pathogenesis of OA. Therefore, we examined the TNF-α level in culture supernatant by enzyme-linked immunosorbent assay (ELISA) (Figure 3G). The above data suggest that knockdown of RIPK1 abolished the inflammatory response induced by administration of IL-1β.

### RIPK1 potentiates TRIF- and myeloid differentiation primary response 88 (MyD88)-dependent IL-β–induced inflammation

**Figure 4:**
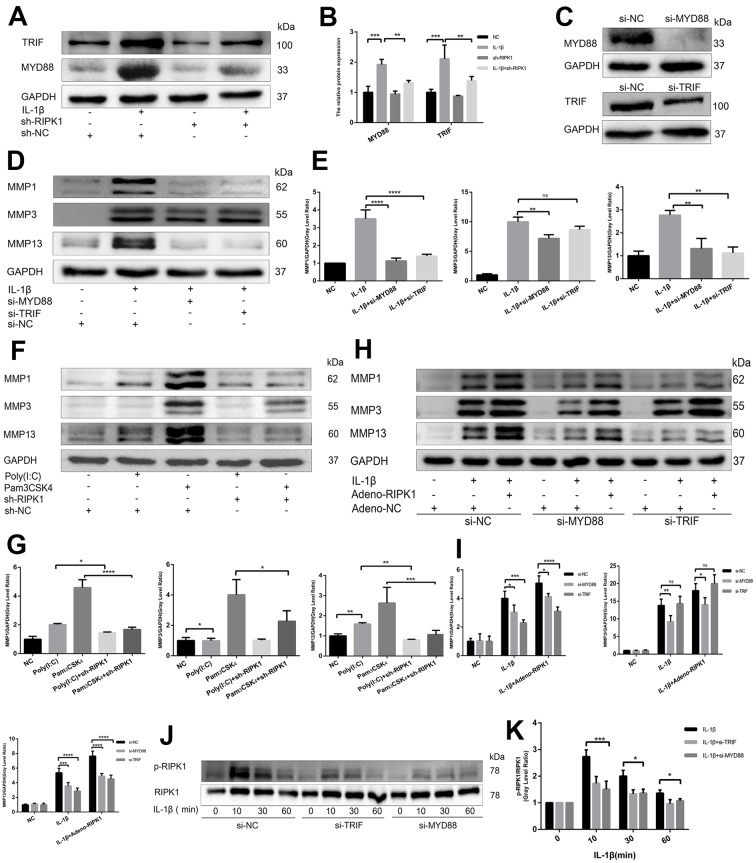
**RIPK1 potentiates TIR-domain-containing adapter-inducing interferon b (TRIF)- and myeloid differentiation primary response 88 (MyD88)-dependent IL-β–induced inflammation.** (**A**, **B**) Western blots and quantitative data of TRIF and MyD88 in chondrocytes transfected with Ad-shRIPK in the presence and absence of IL-1β. © Western blots of TRIF and MyD88 in chondrocytes transfected with si-TRIF or si-MyD88. (**D**, **E**) Western blots and quantitative data of MMPs in chondrocytes transfected with si-TRIF or si-MyD88 in the presence and absence of IL-1β. (**F**, **G**) Western blots and quantitative data of MMPs in chondrocytes incubated with poly (I:C) or Pam3CSK4 in the Ad-shRIPK and Ad-NC groups. (**H**, **I**) Western blots and quantitative data of MMPs in chondrocytes transfected with Ad-shRIPK adenovirus in the si-TRIF and si-MyD88 groups. (**J**, **K**) Western blots and quantitative data of p-RIPK1 in chondrocytes treated with IL-1β in the si-TRIF and si-MYD88 groups. The experiments were repeated three times independently. Columns represent means ± SD. *p < 0.05, **p < 0.01, ***p < 0.001, ****p < 0.0001.

The IL-1 receptor is a member of the TLR superfamily [27]. Engagement of TLRs by IL-1β promotes receptor dimerization and stabilizes recruitment of two essential adaptor proteins, MyD88 and TRIF, to initiate downstream signaling events [28]. To analyze whether the RIPK1 kinase-mediated expression of proinflammatory genes is dependent on MyD88 and TRIF, we analyzed the production of MyD88 and TRIF in the presence and absence of Ad-shRIPK1. The production of MyD88 and TRIF was reduced by RIPK1 knockdown (Figure 4A and 4B). Next, we used Pam3CSK4, an agonist of TLR2 that exclusively engages MyD88, and poly(I:C), an agonist of TLR3 that exclusively engages TRIF, to assess the role of MyD88 and TRIF in inflammation. Knockdown of RIPK1 decreased the Pam3CSK4- and poly(I:C)-induced production of MMPs (with the exception of MMP3) (Figure 4F, 4G). Consistently, knockdown of MyD88 suppressed the IL-1β–induced production of MMPs, and knockdown of TRIF suppressed that of MMP1 and MMP13 (Figure 4D, 4E). Thus, knockdown of RIPK1 relieved MyD88- and TRIF-mediated IL-1β–induced inflammation.

To investigate the relationship between RIPK1 and adaptor proteins (MyD88 and TRIF), MyD88- and TRIF-knockdown chondrocytes were treated with adenovirus overexpressing RIPK1 in the presence or absence of IL-1β. The upregulation of MMPs induced by the overexpression of RIPK1 was decreased in the si-MyD88 and si-TRIF groups (Figure 4H, 4I). Also, the phosphorylation of RIPK1 was decreased compared with the control (Figure 4J, 4K). Overall, these data suggest that RIPK1 potentiates TRIF- and MyD88-dependent IL-β–induced inflammation.

### TRAF2 regulates RIPK1-mediated inflammation

**Figure 5:**
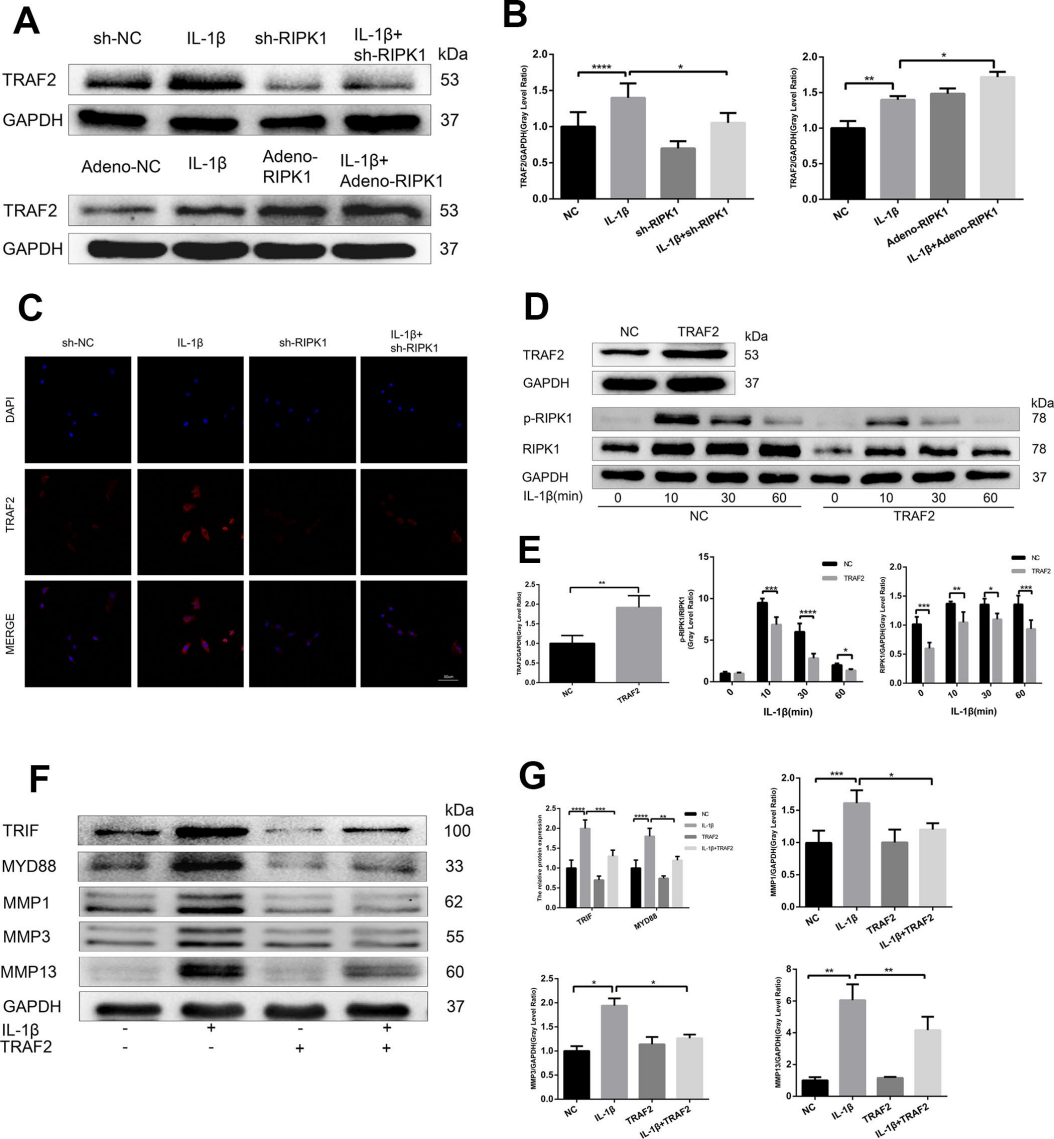
**TRAF2 regulates RIPK1-mediated inflammation.** (**A**, **B**) Western blots and quantitative data of TRAF2 in chondrocytes transfected with Ad-shRIPK and Ad-RIPK1 in the presence and absence of IL-1β. © TRAF2 expression determined by immunofluorescence staining. Scale bar = 50 μm. (**D**, **E**) Western blots and quantitative data of p-RIPK1 and RIPK1. (**F**, **G**) Western blots and quantitative data of TRIF, MYD88, and MMPs. The experiments were repeated three times independently. Columns represent means ± SD. *p < 0.05, **p < 0.01, ***p < 0.001, ****p < 0.0001.

Ian et al. [29] showed that RIPK1 inhibits cell death by interacting with TRAF2 in OA, but the mechanism is unclear. First, we examined TRAF2 expression in chondrocytes. Western blotting revealed that the IL-1β–induced upregulation of TRAF2 was increased and decreased by RIPK1 overexpression and knockdown, respectively (Figure 5A, 5B). Immunofluorescence staining and laser confocal microscopy yielded consistent results (Figure 5C). Next, we overexpressed TRAF2 in chondrocytes to determine whether it modulates RIPK1-mediated OA. As expected, the IL-1β–induced phosphorylation of RIPK1 was significantly reduced by TRAF2 overexpression (Figure 5D, 5E). Furthermore, the increase in the MYD88, TRIF, and MMP levels was similar to that caused by overexpression of TRAF2 (Figure 5F, 5G). Finally, TRAF2 reduced the phosphorylation of RIPK1, suggesting that TRAF2 and RIPK1 form a negative feedback system.

### RIPK1 kinase-mediated osteoarthritis is dependent on apoptosis but not mixed lineage kinase domain like pseudokinase-dependent necroptosis

**Figure 6:**
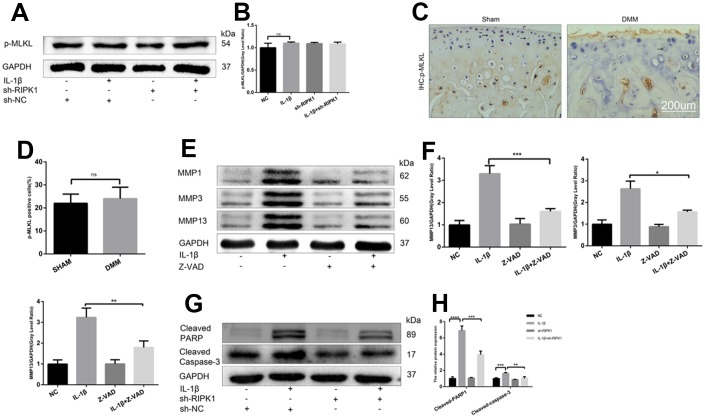
**RIPK1 kinase-mediated osteoarthritis is dependent on apoptosis but not mixed lineage kinase domain-like pseudokinase (MLKL)-dependent necroptosis.** (**A**, **B**) Western blots and quantitative data of p-MLKL. (**C**, **D**) Immunohistochemical staining of p-MLKL in the normal and DMM groups (n = 10); scale bar = 200 μm. (**E**, **F**) Western blots and quantitative data of MMPs. (**G**, **H**) Cleaved caspase 3 and cleaved poly (ADP-ribose) polymerase (PARP) levels in mouse chondrocytes treated as above. The experiments were repeated three times independently. *p < 0.05, **p < 0.01, ***p < 0.001, ****p < 0.0001.

**Figure 7:**
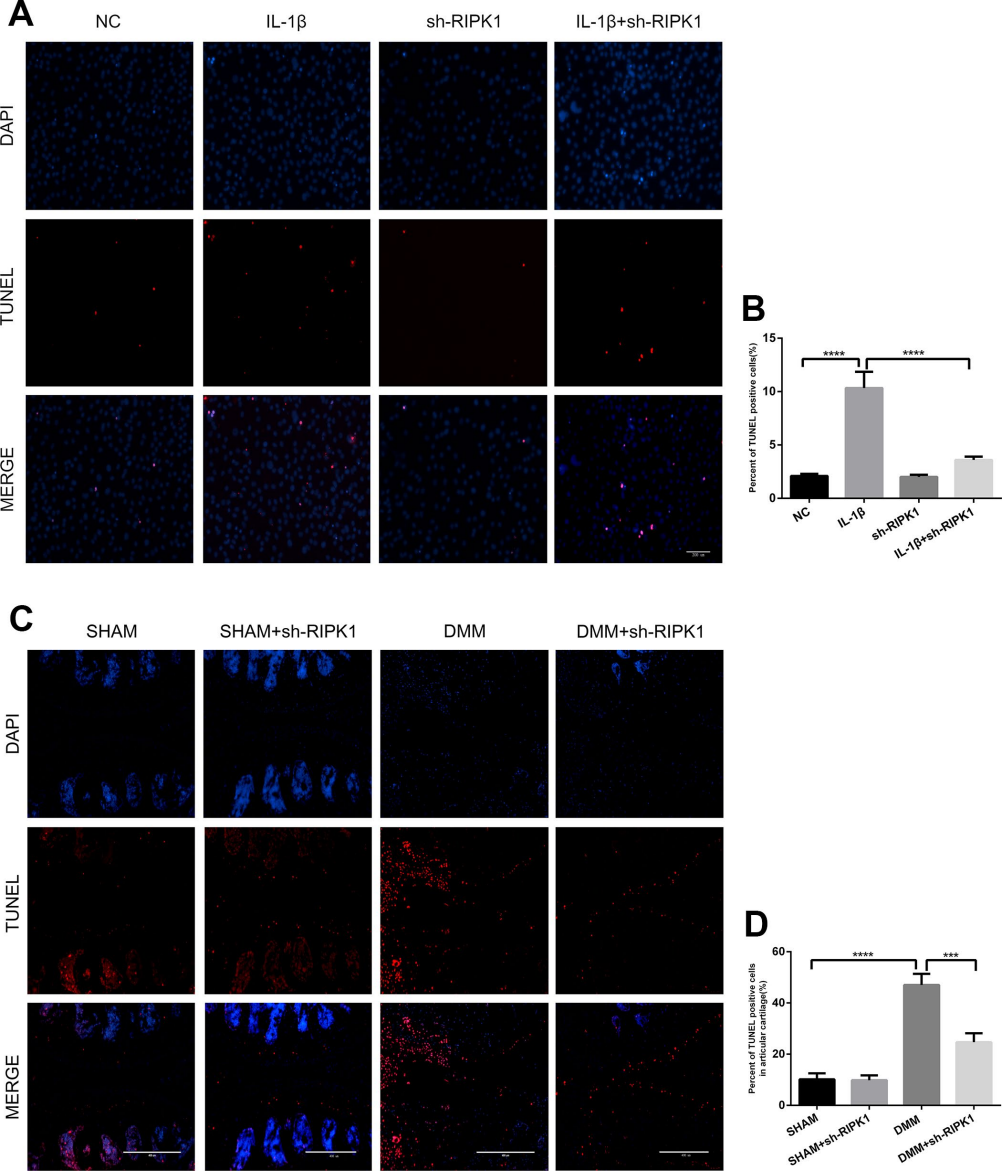
**Knockdown of RIPK1 protects chondrocytes against apoptosis in vitro and in vivo.** (**A**, **B**) Transferase dUTP nick-end labeling (TUNEL)-stained mouse chondrocytes treated as above; scale bar = 200 μm. (**C**, **D**) TUNEL-stained paraffin-wax sections of articular cartilage. Red fluorescence, apoptotic cells; blue fluorescence, nuclei (n = 10). Scale bar = 400 μm. The experiments were repeated three times independently. Columns represent means ± SD. ***p < 0.001, ****p < 0.001.

The ability of RIP1-RIP3 complexes to trigger inflammation is mediated by the necrosis effector protein mixed lineage kinase domain-like pseudokinase (MLKL) and apoptosis effector caspases [30, 31]. We examined the phosphorylation of MLKL, a marker of necroptosis, *in vivo* and *in vitro*. In contrast to previous reports, there was no significant difference in MLKL phosphorylation between the OA and control groups (Figure 6A–6D). Chondrocytes were incubated with IL-1β in the presence or absence of the pan-caspase inhibitor zVAD.fmk (zVAD), and the results showed that the upregulation of MMPs induced by IL-1β was abrogated by zVAD (Figure 6E, 6F). To assess the effect of RIPK1 knockdown on IL-1β–induced apoptosis, the levels of cleaved caspase 3, cleaved poly (ADP-ribose) polymerase (PARP), and DNA damage were measured by Western blotting and terminal deoxynucleotidyl transferase dUTP nick-end labeling (TUNEL) staining, respectively. RIPK1 knockdown reduced the IL-1β–induced increased expression of cleaved caspase 3 and cleaved PARP and the number of TUNEL-positive cells (Figure 6G, 6H and Figure 7A, 7B). TUNEL staining of articular cartilage yielded a similar result (Figure 7C, 7D). In summary, RIPK1 kinase-mediated OA is dependent on apoptosis, but not on MLKL-dependent necroptosis.

### c-Jun N-terminal kinase (JNK) and NF-κB are involved in RIPK1-mediated inflammation

**Figure 8:**
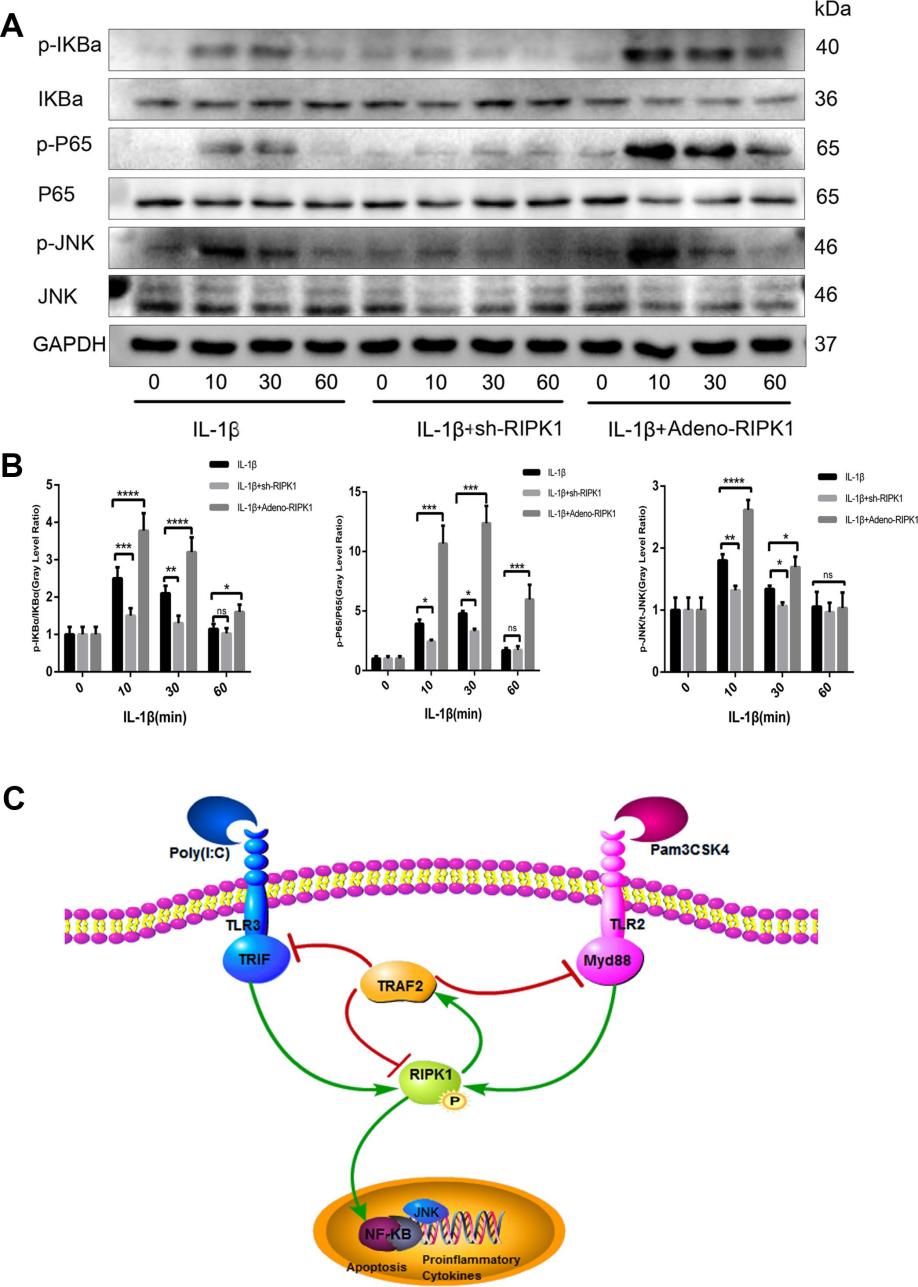
**c-Jun N-terminal kinase (JNK) and nuclear factor-κB (NF-κB) are involved in RIPK1-mediated inflammation.** (**A**, **B**) Expression levels of p-JNK, p-IKBα, and p-P65 in chondrocytes. The experiments were repeated three times independently. Columns represent means ± SD. *p < 0.05, **p < 0.01, ***p < 0.001, ****p < 0.0001. © Schematic of the TIR-domain-containing adapter-inducing interferon b (TRIF)/MYD88-RIPK1-tumor necrosis factor receptor-associated factor 2 (TRAF2) feedback loop and activation of the NF-κB and JNK signaling pathways in OA.

JNK and NF-κB are downstream of the IL-1 signal transduction pathway and play a critical role in OA [32]. To investigate the mechanism by which RIPK1 regulates inflammation, we assayed the phosphorylation levels of components of the JNK and NF-κB pathways in chondrocytes. The phosphorylation of JNK and NF-κB pathway components was reduced in IL-1β–treated chondrocytes transfected with Ad-shRIPK1 (Figure 8A, 8B). However, the opposite result was obtained in chondrocytes transfected with Ad-RIPK1.

## DISCUSSION

OA is mainly driven by proinflammatory cytokines such as IL-1β and TNF-α [33], and is characterized by cartilage damage, ectopic bone formation, and synovitis. RIPK1 interacts with TRADD and TRAFs, which are associated with TLRs [34]. RIPK1 is involved in the pathogenesis of, for example, amyotrophic lateral sclerosis, Alzheimer’s disease, Parkinson’s disease, traumatic brain injury, stroke, and lysosomal storage diseases. We previously reported the effect of a specific inhibitor (NEC-1) of RIPK1 on OA [35]; however, we did not identify the underlying mechanism. In this study, knockdown of RIPK1 attenuated OA *in vivo* and *in vitro* via TRIF and the MYD88-RIPK1-TRAF2 negative feedback loop, and by modulating apoptosis. We generated a DMM by transecting the anterior cruciate ligament of the right knee of the mouse. The phosphorylation of RIPK1 was decreased in the articular cartilage of DMM mice. To explore the role of RIPK1 in OA, chondrocytes were transfected with an adenovirus to induce overexpression of RIPK1 *in vivo* and *in vitro*. The overexpression of RIPK1 inhibited DMM-induced cartilage degeneration, reduced the expression of MMPs, and ameliorated synovitis. The proinflammatory cytokines IL-1β and TNF-α are important in OA because they promote the secretion of other proinflammatory cytokines. IL-1β and TNF-α stimulate chondrocytes to release MMPs, key regulators of cartilage destruction [36]. We used IL-1β to recapitulate the pathogenesis of OA *in vitro* (IL-1β). Primary chondrocytes were transfected with an adenovirus to overexpress or knockdown RIPK1 *in vitro*. These results are consistent with those *in vivo*.

TLRs use the MyD88 and TRIF signaling pathways to promote apoptosis and inflammation [37]. Zhang et al. [38] reported that the induction of MMP production by TLR3 and TLR1/2 or TLR6/2 ligands is dependent on TRIF and MyD88, respectively. Indeed, our data showed that the expression of MyD88 and TRIF was increased by agonists of TLR2 (Pam3CSK4) and TLR3 (poly(I:C)). Also, knockdown of MyD88 and TRIF blocked the Pam3CSK4-, poly(I:C)-, and IL-1β-induced production of MMPs. RIPK1 and RIPK3, but not MYD88, reportedly potentiate TRIF-dependent inflammatory signaling downstream of TLR [39]. In contrast, our data suggest that RIPK1 expression is indispensable for MYD88- and TRIF-dependent production of MMPs in OA. Also, the promotion by RIPK1 of the production of MMPs was dependent on MYD88 and TRIF, as the IL-1β–induced phosphorylation of RIPK1 was blocked by knockdown of MYD88 or TRIF. A proteomics analysis of the interactions between RIPK1 and MYD88/TRIF in chondrocytes incubated with IL-1β is needed to identify the molecular mechanism.

TRAF2 is vital for preventing RIPK1-mediated apoptosis. In addition, TRAF2 rapidly undergoes proteasomal degradation following stimulation of RIPK1-deficient hepatocytes and embryonic fibroblasts via a caspase-independent necrotic cell death mechanism [40]. Also, the physical scaffold of RIPK1 on complex I prevented the proteasomal degradation of TRAF2 [41]. The expression level of TRAF2 was significantly increased by RIPK1 overexpression. Interestingly, the increased expression of TRAF2 blocked the expression and phosphorylation of RIPK1. The mechanism by which TRAF2 modulates TLR signaling is unclear. Vince et al. showed that the NF-κB signaling pathway is required for TLR-induced production of inflammatory cytokines, which is promoted by degradation of TRAF2 [42]. However, Thomas et al. reported that IL-1β significantly increased the expression of TRAF2 in a time-dependent manner [43], which is consistent with our findings. Also, upregulation of TRAF2 decreased the expression of TRIF, MyD88, and MMPs in chondrocytes. RIPK1 is implicated in both apoptotic and necroptotic cell death. Necroptosis, a caspase-independent type of necrotic cell death, is initiated by stimulation with FasL, TNF, or a TNF ligand in the presence of the pan-caspase inhibitor, Z-VAD [44]. RIPK3 mediates the phosphorylation of, and subsequently activates, MLKL, which is required for necroptosis [45]. Additionally, zVAD is required for proinflammatory cytokine-induced accumulation of RIPK1 and phosphorylation of MLKL [46]. In this study, we re-examined the role of necroptosis in IL-1β-induced chondrocytes and DMM cartilage and found that the expression of p-MLKL was similar between the OA and control groups; the pathomechanism mainly involved apoptosis. Also, zVAD reduced the levels of apoptosis and inflammation *in vitro* and *in vivo*. NF-κB signaling is mediated by RIPK1 and inhibits apoptosis and necroptosis by suppressing the expression of c-FLIP and cIAPs [47, 48]. As expected, knockdown of RIPK1 blocked the activation of NF-κB signaling, as evidenced by reduced phosphorylation of p65 and IKBα. JNK signaling was also inhibited by TRAF2 knockdown.

In conclusion, we report that knockdown of RIPK1 in chondrocytes exerts anti-apoptotic and -inflammatory effects *in vivo* and *in vitro*. Also, the regulatory effect of RIPK1 was associated with the TRIF/MYD88-RIPK1-TRAF2 negative feedback loop and the activation of NF-κB and JNK. These results suggest that RIPK1 could be a novel target for the treatment of OA.

## MATERIALS AND METHODS

### Reagents and antibodies

Recombinant murine IL-1b (#211-11B) was purchased from PeproTech (Rocky Hill, NJ, USA). ZVAD was purchased from Selleck Chemicals (Houston, TX, USA). A mouse TNF-α ELISA kit was purchased from Bangyi (Shanghai, China). Murine anti-GAPDH antibody (BM3876) and secondary antibodies were purchased from Boster (Wuhan, China). Antibodies against RIPK1 (#3493), p-RIPK1 (83613), p-JNK (#9255), p-IkBa (#2859), p-P65 (#3033), TRAF2 (#4712), cleaved PARP (#9544), and cleaved caspase-3 (#9964) were purchased from Cell Signaling Technology (Beverly, MA, USA). Rabbit antibodies against JNK (24164-1-AP), IkBa (10268-1-AP), p65 (10745-1-AP), matrix metalloproteinase (MMP) 1 (10371-2-AP), and Myd88 (23230-1-AP) were purchased from Proteintech Group (Wuhan, Hubei, China). Antibodies against p-MLKL (ab196436), TRIF (180619), MMP3 (ab53015), and MMP13 (ab39012) were obtained from Abcam (Cambridge, UK). Pam3CSK4 and Poly (I:C) were obtained from Tocris Bioscience (Bristol, UK). A terminal deoxynucleotidyl transferase-mediated dUTP nick-end labeling (TUNEL) apoptosis detection kit was obtained from Beyotime (Shanghai, China).

### Animals and the OA model

Adult male C57BL/6 mice (age = 12 weeks; mean body weight = 25 g) were used to induce the OA model via DMM surgery on the right knee. Forty mice were divided into four groups: 1) sham group: sham-operated mice administered Ad-negative adenoviruses (n = 10); 2) the sham + Ad-shRIPK1 group: sham-operated mice treated with Ad-shRIPK1 adenoviruses (n = 10); 3) the DMM group: DMM-operated mice administered Ad-negative adenoviruses (n = 10); and 4) the DMM + Ad-shRIPK1 group: DMM-operated mice administered Ad-shRIPK1 adenoviruses (n = 10).

Briefly, after anesthetization, the anterior fat pad was excised to expose the anterior medial menisco-tibial ligament, which was then transected. In the control group, a sham operation was performed in which only the anterior fat pad was excised [49]. After wound healing, intra-articular injection of 10 μL Ad-shRIPK1 or Ad-negative adenoviruses (1 × 10^9^ plaque forming units [PFUs]) was administered to the mice once a week for 8 weeks [50]. The animal experiment was approved by the Ethics Committee on Animal Experimentation of Tongji Hospital.

### Adenovirus and plasmids

The adenoviral vectors carried GFP, mouse RIPK1, and RIPK1 shRNA, and were designed by Vigene Biosciences (Shandong, China). The shRNA sequence targeting the mouse RIPK1 sequence (GenBank No. NM_009068) was designed as follows: GCAGAGAGC TCGTGAGAATATTCAAGAGAATATTCTCACGAGC TCTCTGCTTTTTT. The cells were transfected with Ad-shRIPK1 and Ad-negative adenoviruses at a confluence of 70%. The medium was changed after 12 h and the cells were incubated for a further 2 days. According to the observed fluorescence intensity of GFP, the multiplicity of infection (MOI) was about 50:1. DDK-tagged TRAF2 and the control vector were purchased from OriGene Technologies (Rockville, MD, USA).

### Histological staining and analysis

The right knee joint samples were fixed in 4% paraformaldehyde for 48 h and decalcified with EDTA-buffered saline solution for 15 days. Tissue sections were then embedded in paraffin wax and cut into 4-μm-thick slices in the sagittal plane for hematoxylin and eosin (HE) and Safranin O staining. The level of knee joint degeneration was measured using the Osteoarthritis Research Society International (OARSI) scores [51] and arbitrary scale [52]. The levels of RIPK1, MMP1, MMP3, MMP13, and p-MLKL were evaluated in each group using an immunohistochemical staining kit (DAB Kit, Invitrogen, Paisley, UK). Images were captured under a digital microscope (Nikon ECLIPSE Ti-S, Nikon, Tokyo, Japan) and analyzed using ImageJ software (NIH, Bethesda, MD, USA).

### TUNEL staining

TUNEL staining was used to detect apoptosis in each group of chondrocytes and cartilage. After fixation in 4% paraformaldehyde, chondrocytes or cartilage sections were stained with the TUNEL apoptosis detection kit and DAPI at 37°C according to the manufacturer’s instructions. The images were captured under a fluorescence microscope (Nikon ECLIPSE Ti-S, Nikon, Tokyo, Japan) and TUNEL-positive cells were analyzed using ImageJ software.

### Cell culture

Mouse primary chondrocytes were extracted from the bilateral knee joint cartilage of newborn C57BL/6 mice as described previously [53]. Briefly, hyaline cartilage was cut into pieces and incubated with 0.25% trypsin-EDTA for 30 min and placed in 0.2% type II collagenase for 8 h at 37°C. After resuspension and filtration, the released cells were cultured in DMEM/F12 media supplemented with 10% fetal bovine serum (FBS), 100 U/mL penicillin, and 100 mg/mL streptomycin sulfate in 25 cm^2^ flasks at 37°C under 5% CO_2_. To preserve the chondrocyte phenotype, only cells from the first or second passage were used in the experiments.

### siRNA transfection

siRNA targeting TRIF and MYD88 were transfected into primary chondrocytes to knock down the expression of the targeted mRNA according to the manufacturer’s instructions (Ribbio, Guangzhou, China). The chondrocytes were transfected with 50 nM siRNA duplexes using Lipofectamine 3000 siRNA transfection reagent (Invitrogen, Carlsbad, CA, USA). The siRNA sense strands were as follows: (TRAF2) 5′-GTCTACGA GTCTACTTGAA-3′; (MYD88) 5′-GACTGATTCCTAT TAAATA-3′; (TRIF) 5′-TCTTGTTACTGACTGAGAA-3′. The efficiency of the targeted gene silencing was confirmed by western blotting.

### Immunofluorescence and confocal microscopy

A total of 50 000 cells were planted on a culture slide (Corning, Corning, NY, USA) to facilitate confocal microscopic observation. After fixation in 4% paraformaldehyde, chondrocytes were incubated in 0.2% Triton X-100 for 10 min and blocked with 5% bovine serum albumin for 30 min. Then, the chondrocytes were incubated overnight at 4°C with primary rabbit anti-mouse TRAF2 antibody. Afterward, the cells were incubated with secondary goat anti-rabbit Cy3-conjugated antibody (Invitrogen, Camarillo, CA, USA) for 1 h at 25°C. Finally, culture slides were stained with 4, 6-diamidino-2-phenylindole (DAPI) for 10 min and washed with PBS in the dark. Immunofluorescence was imaged with a confocal laser scanning microscope (Leica Microsystems CMS GmbH, Mannheim, Germany).

### Western blot analysis

The total protein extracted from chondrocytes was isolated using ice-cold lysis buffer with 1% protease and phosphatase inhibitors for 30 min, followed by centrifugation at 12 000 rpm and 4°C for 20 min. The BCA protein assay kit (Boster) was used to measure the protein concentration. A total of 25 ng of protein was loaded onto SDS-PAGE gels (10–15%) and then transferred to polyvinylidene fluoride membranes (Millipore, Billerica, MA, USA). Membranes were blocked with 5% BSA for 1 h and incubated with primary antibody overnight at 4°C, followed by addition of HRP-conjugated secondary antibodies for 1 h. Finally, the signal was visualized using Pierce ECL Western Blotting Substrate in the ChemiDoc XRS System (Bio-Rad Laboratories, Hercules, CA, USA).

### Statistical analysis

The results are presented as means ± SD. Statistical analyses were performed using Graphpad Prism v. 5.0 (Graphpad Software Inc., San Diego, CA, USA). Student’s *t*-test was used to assess differences between two groups, and one-way analysis of variance (ANOVA) followed by Dunnett’s post hoc test was used to compare groups. All of the cell experiments were performed with at least three independent biological replicates. *P*-values < 0.05 were considered significant.
